# Expanding the Spectrum of *EWSR1-NFATC2*-rearranged Benign Tumors: A Common Genomic Abnormality in Vascular Malformation/Hemangioma and Simple Bone Cyst

**DOI:** 10.1097/PAS.0000000000001748

**Published:** 2021-06-03

**Authors:** Sheena L.M. Ong, Suk Wai Lam, Brendy E.W.M. van den Akker, Herman M. Kroon, Inge H. Briaire-de Bruijn, Arjen H.G. Cleven, Dilara C. Savci-Heijink, Anne-Marie Cleton-Jansen, Daniel Baumhoer, Karoly Szuhai, Judith V.M.G. Bovée

**Affiliations:** Departments of * Pathology; † Radiology; ∥ Cell and Chemical Biology, Leiden University Medical Center, Leiden; ‡ Department of Pathology, Amsterdam UMC, location AMC, Amsterdam, The Netherlands; § Department of Medical Genetics and Pathology, Bone Tumor Reference Center, University Hospital Basel and University of Basel, Basel, Switzerland

**Keywords:** simple bone cyst, *EWSR1-NFATC2*, vascular malformation, bone tumor, hemangioma

## Abstract

A simple bone cyst (SBC) is a cystic bone lesion predominantly affecting young males. The cyst is lined by a fibrous membrane and filled with serosanguinous fluid. *EWSR1/FUS-NFATC2* rearrangements were recently identified in SBC. We here report exactly the same rearrangement in 3 lesions diagnosed as vascular malformations of 2 elderly patients. In total, through Archer FusionPlex, fluorescence in situ hybridization and/or reverse transcriptase-polymerase chain reaction the *EWSR1-NFATC2* rearrangement was identified in 6 of 9 SBC, 3 of 12 benign vascular tumors, and none of 5 aneurysmal bone cyst lacking *USP6* fusion. Using fluorescence in situ hybridization, it was apparent that amplification of the fusion, as seen in *EWSR1-NFATC2* round cell sarcomas, was absent, and that in the vascular tumors the fusion was present both in the lining cells as well as in the surrounding spindle cells. Of note, not all of the spaces in the vascular malformations were lined by endothelial cells. Aggrecan was positive in all cases but was not specific. NKX2-2 and NKX3-1 staining were negative in all cases. Thus, even though the overlap between the 2 entities is limited to the presence of few thick-walled cysts lacking endothelial lining in the benign vascular malformations, the spectrum of benign tumors containing *NFATC2* fusions should be expanded and contains not only SBC in the young, but also vascular malformation/hemangioma in elderly patients.

## Abstract 2

Supplemental Digital Content is available in the text.

Rearrangements involving *EWSR1* or *FUS* and *NFATC2* were recently shown to characterize simple (solitary) bone cyst (SBC).^1^ Two separate series have been reported and of 18 cases that were successfully analyzed, 11 cases (∼60%) showed *NFATC2* rearrangement with either *FUS* (6/11) or *EWSR1* (5/11).^1^,^2^ SBC is an intramedullary, usually unilocular, cystic bone lesion lined by a fibrous membrane and filled with serosanguinous fluid.^3^ This lesion predominantly affects the long tubular bones during the first 2 decades of life and is more common in males. The lesions can be asymptomatic, cause pain or swelling, or cause a pathologic fracture. Histologically, fibrous septations can be seen representing the wall of the cyst, with foci of chronic inflammation, multinucleated giant cells, myxoid change, and reactive bone. Typical fibrin-like collagen deposits are common. The lesions are not reported to have any specific immunohistochemical profile and the diagnosis is usually not difficult and made based on hematoxylin and eosin in correlation with clinical and radiologic findings.

The finding of *EWSR1*/*FUS*-*NFATC2* rearrangements in SBC is remarkable as the same alterations have been reported previously to characterize a small blue round cell sarcoma, at the time referred to as Ewing-like sarcoma.^3^,^4^ Currently, the group of undifferentiated small round cell sarcomas of bone and soft tissue contains 4 entities; (a) Ewing sarcoma (characterized by fusions involving one member of the FET family of genes [*EWSR1*/*FUS*] and a member of the ETS family of transcription factors), (b) round cell sarcoma with *EWSR1*/*FUS* gene fusion with non-ETS family members (which includes *NFATC2*), (c) *CIC*-rearranged sarcoma, and (d) sarcoma with *BCOR*-genetic alterations.^3^ Round cell sarcoma with *EWSR1*/*FUS*-*NFATC2* fusion manifests as a locally destructive bone lesion that may extend into the soft tissue, with a wide age range (12 to 67 y), and a strong male predominance. *EWSR1/FUS*-*NFATC2* round cell sarcomas are composed of undifferentiated small to medium-sized round cells, with limited cytoplasm. These cells can be arranged in cords or small nests and the stroma can be prominent. Necrosis and mitoses can be observed. Immunohistochemically, the tumor cells can show diffuse staining for CD99 in ∼50% of the reported cases.^3^ NKX2-2 and PAX7 can also be expressed^5^,^6^ and dot-like keratin or epithelial membrane antigen (EMA), focal WT-1, CD138, SATB2, p63, and smooth muscle actin (SMA) staining can be observed in a subset.^5^,^7^,^8^ Desmin, S100, synaptophysin, and chromogranin are negative.^4^,^7^–^10^ Thus, even though *NFATC2* rearranged round cell sarcoma and SBC share the same fusion gene, any morphologic and immunohistochemical overlap is absent and their clinical behavior is entirely different.

More recently, 2 novel markers were identified for *EWSR1*-*NFATC2* round cell sarcomas: NKX3-1 was reported to be moderately or strongly expressed in 82%, while other round cell tumors and a single case of *FUS*-*NFATC2* sarcoma were negative.^11^ Expression was subsequently confirmed in 3 cases using a monoclonal antibody as well as at the RNA level.^12^ In contrast, Perret et al^9^ reported focal or diffuse staining of aggrecan in all cases, while NKX3-1 was negative in all 6 *NFATC2*-rearranged round cell sarcomas tested.^9^ Nothing is known about the expression of aggrecan in SBC, while 2 cases of SBC were negative for NKX2-2 and NKX3-1.^2^ Of note, expression of CD99, SMA, and EMA was recently reported in SBC.^1^

We here report that the same *EWSR1*-*NFATC2* rearrangement is also recurrent in benign vascular tumors. In addition to a series of SBCs occurring in the long bones of young patients, we describe 2 elderly patients presenting with vascular malformations carrying *EWSR1*-*NFATC2* rearrangement, which were multifocal in 1 patient. There was only very limited morphologic overlap with SBC. We show the presence of the fusion in both the lining/endothelial cells as well as the surrounding spindle cells. In addition, we evaluate the expression of recently reported markers (including aggrecan, NKX3-3, and NKX2-2). This way we further expand the clinical spectrum of *EWSR1*-*NFATC2*-rearranged tumors.

## MATERIALS AND METHODS

### Case Selection

Two tumors with *EWSR1*-*NFATC2* rearrangement were encountered in our routine consultation practice with the different clinical and histopathologic presentations. One rib tumor displayed the morphology of a vascular malformation (L6829), while the other was a cystic lesion in the femur of a young child (L6831). On the basis of the morphologic variation we proceeded onto an extensive query search from the archives of Leiden University Medical Center (LUMC), Leiden for patients diagnosed with either SBC, aneurysmal bone cyst (ABC) lacking *USP6* rearrangement, hemangioma of bone, or vascular malformation of bone. Vascular malformation cases in either bone or soft tissue were also retrieved from the archives of the Bone Tumor Reference Center in Basel, Switzerland. Including the 2 index cases, 7 ABC of 7 patients, 12 SBC of 12 patients, and 18 benign vascular tumors of 16 patients were investigated. The benign vascular tumors included vascular malformations (admixed thick and thin-walled large and small caliber vessels) (n=10) as well as hemangioma of bone (thin-walled vessels in the medulla of bone at typical locations in axial skeleton) (n=7). In addition, one of the index patients also had a spindle cell hemangioma previously. Detailed information of all 37 cases (35 patients) is listed in Supplementary Table 1 (Supplemental Digital Content 1, http://links.lww.com/PAS/B162). Surgical pathology specimens were routinely decalcified with formic acid. Frozen tissue was available for 15 patients. Slides were reviewed by experienced bone tumor pathologists (J.V.M.G.B., A.H.G.C., or C.D.S.H.). All samples were handled according to the ethical guidelines described in “Code for Proper Secondary Use of Human Tissue in the Netherlands” in a coded (pseudonymized) manner, as approved by the Leiden University Medical Center Ethical Board (B20.025).

### Fluorescence In Situ Hybridization

Formalin-fixed paraffin-embedded (FFPE) tissues were cut on DAKO glass slides (Agilent, S3003) with 3 to 5 µM thickness. Fluorescence in situ hybridization (FISH) was performed using the Histology FISH accessory kit according to standard procedure (Agilent, K5799). Two types of the probe were utilized: either Kreatech EWSR1-NFATC2 t(20;22) dual-color single-fusion FISH probe for the detection of colocalization of translocated regions of *EWSR1* and *NFATC2* (Leica, KBI-10751) or using a split apart in-house designed bacterial artificial chromosome probe set: RP5-994O24 and RP5-1114A1 bracketing the proximal and distal regions of *NFATC2* on chromosome 20, respectively, using an indirect detection system as described in detail by Szuhai and colleagues. Images were scanned using 3DHISTECH Pannoramic scannerP250 (3DHistech, Hungary) then visualized and scored in CaseViewer, version 2.3.0.99276 (3DHistech). The total number of nuclei counted varied from 43 cells to 431 depending on size and/or quality of the material. Translocation cases were considered positive when a translocation event was detected in at least 20% of the total nuclei counted.

### Reverse Transcriptase-Polymerase Chain Reaction

RNA was isolated from frozen tissue, available for 8 of the 37 cases, using TRIzol (Ambion, 15596018), chloroform extracted, isopropanol precipitated, and resuspended in RNase-free water. Purified RNA samples were then DNase treated (Qiagen, 29254) and column purified (Qiagen, 74104). AMV reverse transcriptase (Roche, 109118) was used to synthesize 1 µg of total RNA into cDNA. Before real-time PCR (Bio-Rad, 1708886), cDNA samples were diluted 5× with milliQ water. Samples were run in technical duplicates. Primers used for EWSR1-NFATC2 and GAPDH expression are 5′-CAACCTCAATCTAGCACAGG-3′, 3′-AGTCCCAGAGGCTTGTT-5′ and 5′-TTCCAGGAGCGAGATCCCT-3′, 3′-CACCCATGACGAACATGGG-5′, respectively.

### Anchored Multiplex Polymerase Chain Reaction FusionPlex

Cases that failed FISH were not processed for anchored multiplex polymerse chain reaction (AMP) analysis. The AMP system was used to amplify target-enriched cDNA libraries from FFPE and frozen tissues isolated RNA as described before.^13^ Complementary DNA (cDNA) synthesis of first-strand and second-strand was performed and a library of targeted cDNA was generated using Ion Torrent Archer FusionPlex Sarcoma kit v1 (Archer, Boulder, CO). This assay targets and identifies fusions of 26 genes associated with soft tissue tumors including *EWSR1* and *FUS*. cDNA synthesis, thermal cycler settings, and sample reaction for each amplification step were carried out according to Archer FusionPlex Protocol for Ion Torrent. The readouts were analyzed using Archer Analysis software, version 6.2.3 (ArcherDX). The percentage of fusion reads were calculated based on the number of unique reads spanning the breakpoints and supporting the event, divided by the total number of unique reads that span either breakpoint. The quality of the fusion reads was assessed based on the following criteria; quantification cycle of real-time quantitative PCR (QC score) <32, a minimal total read number of 1.5 million a Fusion QC of >10 and >40% RNA reads as advised by the technical support of Archer.

### Immunohistochemistry

Immunohistochemistry for all antibodies except aggrecan was performed using standard diagnostic procedures using the Omnis autostainer (Dako, Agilent). Depending on the type of antibody, antigen retrieval was carried out in either Tris-EDTA pH 9.0 or citrate pH 6.0. Details of antibodies dilutions, use of linkers, and other information are listed in Supplementary Table 2 (Supplemental Digital Content 2, http://links.lww.com/PAS/B163). For aggrecan, 4-µm deparaffinized FFPE sections were rehydrated and endogenous peroxidase blocked. Antigen retrieval was performed with citrate, pH 6.0. Tissue sections were then incubated with antibody overnight at 4°C in 1:16,000 dilution. Detection was applied using immunologic Poly-HRP-GAM/R/IgG (VWR, DVPO110HRP) and Dako liquid DAB+ Substrate Chromogen System (K3468) as described.^14^ Slides were counterstained with hematoxylin. Round cell sarcomas with molecularly proven *EWSR1-NFATC2* rearrangement, previously described as index cases, located in the femur of 16 years old male (L1857) and the humerus of 36 years old male (L1231) were used as control.^4^

## RESULTS

### NFATC2 Fusions Are Common in SBC and Vascular Malformation/Hemangioma

From the 12 SBC cases, 6 were found to carry an *NFATC2* fusion (Table 1), while these fusions were absent in the ABCs that lacked *USP6* rearrangement. For 3 SBCs and 1 ABCs, the material was insufficient for molecular analysis, probably due to prolonged decalcification. In 4 of the 6 SBC cases, the fusion partner was *EWSR1*. For the other 2, *NFATC2* rearrangement was shown by FISH, but RNA isolation failed and there was no tissue left to do FISH for *EWSR1* and/or *FUS*, so the fusion partner remains unknown.

**TABLE 1:** Clinical and Molecular Features of *EWSR1*-*NFATC2* Cases

Case No.	Case ID	Original Diagnosis	Age/Sex	Tumor Location	Tumor Size	Archer (Breakpoints) (% of Reads)	FISH (Fusion Positive %)	RT-PCR (Breakpoints)
1	L6829	Vascular malformation	Female/63	Costa 5 anterior	5 cm	EWSR1-NFATC2 (exon 7—exon 3) (19%)	EWSR1-NFATC2 (49.4%)	EWSR1-NFATC2 (exon 7—exon 3)
	L6952			Sacrum lesion	4.5 cm	No cDNA measured	EWSR1-NFATC2 (53.2%)	NP
	L6947			Foot lesion	1.6 cm	No cDNA measured	failed	NP
2	L6959	Vascular malformation	Male/50	Retrobulbar soft tissue	3.0 cm	No cDNA measured	EWSR1-NFATC2 (36%)	NP
3	L6830	SBC	Male/8	Femur	Maximal diameter: 4.5 cm	EWSR1-NFATC2 (exon 6—exon 3) (40%)	NFATC2 split (20.2%)	No amplifcation
4	L6831	SBC	Male/15	Femur	Maximal diameter: 5 cm	EWSR1-NFATC2 (exon 6—exon 3) (8%)	Failed	EWSR1-NFATC2 (exon 6—exon 3)
5	L6861	SBC	Male/9	Femur	2.5 cm at presentation, later 10 cm	EWSR1-NFATC2 (exon 6—exon 3)	EWSR1-NFATC2 (39.7%)	NP
6	L6863	SBC	Male/8	Femur	Unknown	No cDNA measured	NFATC2 split (24.4%)	NP
7	L7186	SBC	Male/9	Femur	7 cm	EWSR1-NFATC2 (exon 5—exon 3)	—	NP
8	L6837	SBC	Female/29	Finger proximal phalanx digit 4	Maximal diameter: 2 cm	No cDNA measured	NFATC2 split (51.2%)	NP

NP indicates not performed.

**FIGURE 1:**
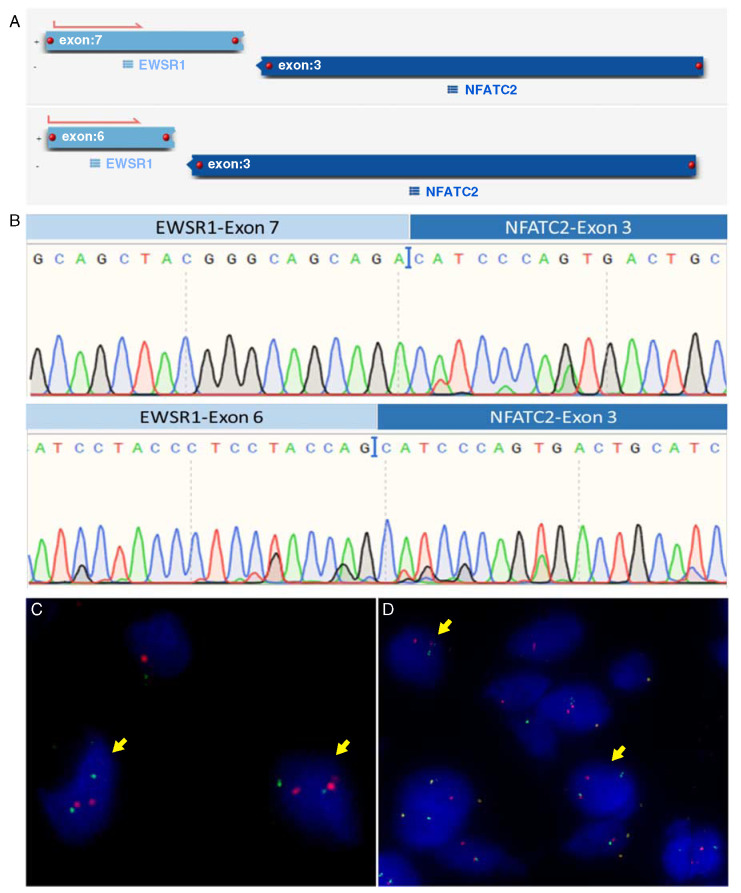
Molecular analysis revealed *EWSR1-NFATC2* fusion. Output of Archer analysis (A) and Sanger sequencing of RT-PCR revealing *EWSR1* (exon 7)—*NFATC2* (exon 3) (up) *EWSR1* (exon 6)—*NFATC2* (exon 3) (bottom) translocations (B). FISH analysis of SBC cases showing *NFATC2* split identified in case 3 (C) and *EWSR1*-*NFATC2* colocalization in case 5 (D). Yellow arrows marking fusion cells.

Of the 16 patients with benign vascular tumors, 2 showed the presence of *EWSR1-NFATC2* rearrangement in their tumors. For 5 patients, the molecular analysis failed mostly due to prolonged tissue decalcification procedure. A summary of the molecular findings of the positive cases is shown in Table 1. Using AMP-based targeted NGS (Archer) for fusion analysis of 5 patients, we identified an *EWSR1* (exon 7; NM_001163285)—*NFATC2* (exon 3; NM_012340.4) fusion in case 1, an *EWSR1* (exon 6; NM_005243.3)—*NFATC2* (exon 3; NM_012340.4) in cases 3, 4, and 5 and an *EWSR1* (exon 5; NM_005243.3)—*NFATC2* (exon 3; NM_012340.4) fusion in case 7 (Fig. 1A). The percentage of fusion reads was above the minimal quality control as defined above. RNA was isolated from frozen tissue of 8 cases for reverse transcriptase-polymerase chain reaction (RT-PCR) amplification spanning the region between *EWSR1* exon 6 (NM_001163285) and *NFATC2* exon 4 (NM_005243.3). Among the 8 cases, only 2 cases revealed amplification of the fusion transcript using RT-PCR confirmed by subsequent Sanger sequencing (Fig. 1B) (Supplementary Table 1, Supplemental Digital Content 1, http://links.lww.com/PAS/B162). FISH was carried out with either *NFATC2* split (Fig. 1C) or *EWSR1*-*NFATC2* (Fig. 1D) colocalization probes and was successful in 7 tumors, confirming *NFATC2* rearrangement. No amplification of the signals was seen. The percentage of fusion nuclei was calculated and ranges from 20.2% to 53.2%.

### EWSR1-NFATC2-rearranged Vascular Malformation/Hemangioma

#### Case 1

**FIGURE 2:**
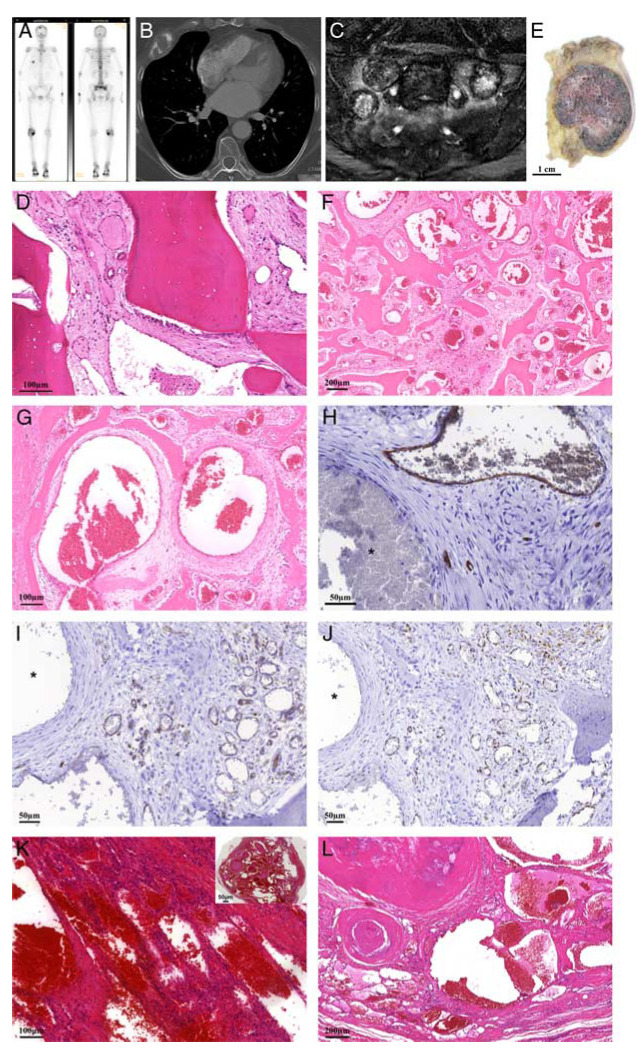
Case 1. A 63-year-old female with 2 benign vascular tumors of bone containing *EWSR1*-*NFATC2* fusion. A, Bone scintigraphy with increased uptake of the tracer in the lesion in the rib, but also, focal abnormalities in the skull, spine and sacrum. B, CT image of the chest with an expansile osteolytic lesion in the fifth rib on the right side. C, Coronal T2-weighted MR image with fat suppression. Multiple lesions in the sacrum with variable signal intensities from predominantly low, as the one in the center, to mixed low and higher and high. Surrounding perilesional edema. D, Hematoxylin and eosin staining of biopsy of the sacral mass shows a tumor mainly composed of thin-walled vessels with no atypia of the lining cells. E, Gross specimen of the rib lesion showing a well-demarcated red tumor in the medullary cavity of the ventral part of the rib with cortical expansion. Low (F) and higher (G) power view of hematoxylin and eosin staining showing that the rib tumor is mainly composed of large dilated blood vessels of variable caliber, some of which are thin walled, while others have a thicker wall, lying within preexisting trabecular bone. Vascular markers CD34 (H), CD31 (I), and ERG (J) confirmed the endothelial lining in only a subset of the spaces, while some of the cysts containing a thicker wall are negative (asterix). Note the background of loose myxoid to more fibrous stroma with bland stromal cells. K and L, Hematoxylin and eosin staining of the vascular lesion in the soft tissue of her foot 22 years previously demonstrating a sharply defined lesion (inset) with different morphology containing mainly numerous large dilated, thin-walled vessels containing prominent phleboliths, admixed with focal spindle cell areas.

A 63-year-old female patient presented with pain on the chest at the Cardiology Department, without any other complaints. Diagnostic workup revealed multiple skeletal lesions, as demonstrated by bone scintigraphy showing increased uptake of the tracer in the rib, the spine, the skull, and pelvis (Fig. 2A). On positron emission tomography-computed tomography (CT) the lesions were mildly fluorodeoxyglucose avid. A conventional radiograph of the skull showed a well-defined osteolytic lesion with a central area of sclerosis. A conventional chest radiograph followed by CT demonstrated an expansile predominantly osteolytic lesion in the fifth rib on the right anterior side (Fig. 2B). CT study of the pelvis demonstrated lesions in the sacrum, iliac wings, pubic bone on the left and right ischial bone. These lesions were partially osteolytic and osteoblastic. On an magnetic resonance (MR) of the sacrum, the masses had a low to intermediate signal intensity on T1-weighted images and mixed low to intermediate to high on T2-weighted images, and surrounding bone marrow edema was visualized (Fig. 2C). Mild to marked enhancement was observed after contrast administration. Radiologic differential diagnostic considerations were metastases of an unknown primary tumor, brown tumors in hyperparathyroidism, and Langerhans cell histocytosis despite the rather advanced age of the patient. Subsequent biopsy of the rib was nonrepresentative, while the biopsy of the sacral mass showed a marked increase of vessels between the bony trabeculae (Fig. 2D) (L6952). Most of these vessels where thin-walled in a notable background of loose myxoid to more fibrous stroma with bland stromal cells. Focally, thick-walled vessels were present suggestive of a vascular malformation. Since based on the multifocality and the radiologic findings there was uncertainty about the diagnosis, a diagnostic resection of the rib was performed (L6829).

Evaluation of the gross specimen (Fig. 2E) revealed a sharp demarcated red to brown nodule of 4×3×5.5 cm, located in the medullary cavity of the ventral part of the rib. Cortical expansion with cortical destruction and soft tissue extension were observed. Resection margins were free from tumor. Histologic examination showed a well-circumscribed, noncapsulated lesion, mainly composed of large dilated blood vessels of variable caliber lying among preexisting trabecular bone (Fig. 2F). Blood vessels were lined by one layer of flattened, bland, and mitotically inactive cells. Most vessels showed a thin wall (Fig. 2G). Nuclear atypia and mitoses were absent. Very focally, smaller vessels with a more lobular architecture could be appreciated. Here, the endothelial cells showed a somewhat more abundant cytoplasm. No atypia was present. Immunohistochemistry for CD31, CD34, and ERG confirmed endothelial differentiation of the lining cells, although some of the spaces that showed a thicker wall were entirely negative (Figs. 2H–J, Table 2). At the background, reactive changes such as hemorrhage with hemosiderin-laden macrophages and reactive woven bone were seen. On the basis of morphology, including the admixture of small and large caliber vessels, a vascular malformation was diagnosed. Both the rib as well as the sacral tumor were shown to carry an *EWSR1-NFATC2* fusion (Table 1).

**TABLE 2:** Immunohistochemical Staining

Case No.	Case ID	CD31	CD34	ERG	EMA	CD99	SMA	NKX2-2	NKX3-1	Aggrecan
1	L6829	Weak	Mosaic†	Mosaic	−	Weak*	Mosaic	−	−	Mosaic
	L6952	+	Mosaic	+	−	+*	+	−	−	Focal
	L6947	Focal	Mosaic	+	−	−	Focal strong	−	NP	NP
2	L6959	Mosaic	+	Weak	Focal	Focal	+	−	−	Mosaic
3	L6830	−	−	−	−	+	+	−	−	+
4	L6831	−	−	−	Focal	Focal weak	Focal	−	−	+
5	L6861	−	−	−	+	+	+	−	−	Focal
6	L6863	NP	NP	NP	NP	NP	NP	NP	NP	NP
7	L7186	−	−	−	+	+	−	−	−	NP
8	L6837	−	−	−	−	+	NP	NP	NP	Focal
9	L1857	Focal weak	−	−	Focal weak	+	−	Focal weak	Focal weak	+‡
10	L1231	−	−	−	+	+	−	Focal weak	Focal weak	+‡

* Lining cells and stromal cells.

† Some larger cysts are entirely negative.

‡ Diffuse.

− indicates negative staining; +, positive staining; NP, not performed.

Interestingly, her medical record revealed a third vascular tumor, not in bone but in the soft tissue of the foot, resected 22 years earlier. The morphology was revised (L6947) but was slightly different from the lesions in the sacrum and the rib, and was suggestive of a spindle cell hemangioma, with numerous large dilated, thin-walled vessels lined by nonatypical endothelial cells, containing prominent phleboliths, admixed with focal spindle cell areas (Figs. 2K, L). Unfortunately, the molecular analysis failed. Three years later, the patient died of an unrelated cause.

#### Case 2

**FIGURE 3:**
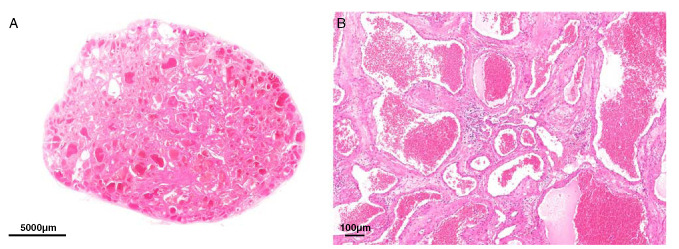
Case 2. A 50-year-old male with retrobulbar vascular malformation containing *EWSR1*-*NFATC2* fusion. A, The lesion is well-defined consisted of haphazardly arranged blood-filled spaces. B, Spaces are lined by a thin layer of endothelial cells. The vessel walls appeared uniformly thin with only few layers of smooth muscle cells, the lining cells lacked cytologic atypia and mitotic activity.

A 50-year-old male patient presented with progressive exophthalmos on the left side without significant visual impairment. MR image showed a well-delineated retrobulbar mass in the soft tissue measuring 3 cm in largest diameter which was subsequently excised with clear margins. Histologically, the lesion consisted of haphazardly arranged blood-filled spaces that were lined by a thin layer of endothelial cells (Fig. 3A). In greater magnification, the vessel walls appeared uniformly thin with only a few layers of smooth muscle cells, the lining cells lacked cytologic atypia and mitotic activity (Fig. 3B). Immunohistochemistry confirmed the endothelial differentiation (CD31 positive) and proliferative activity (MIB-1) was very low except for a few inflammatory infiltrates. The diagnosis of vascular malformation was made and the patient remained in clinical controls for >18 years without showing evidence of recurrent disease.

### *NFATC2*-rearranged SBCs

**FIGURE 4:**
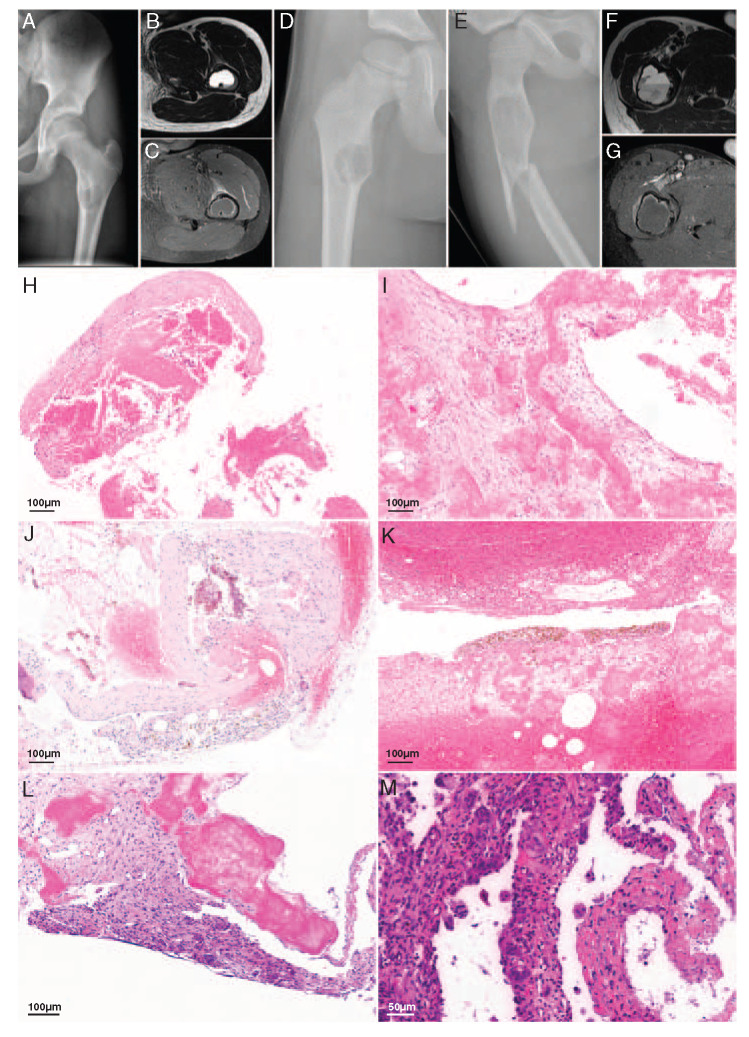
SBCs with *EWSR1*-*NFATC2* fusion. A, Radiologic images of case 4 showed conventional radiograph of the left hip demonstrating a well-defined osteolytic lesion in the left femur. B, Axial T2-weighted MR image with very high signal intensity and a fluid levels on the dependent side. C, Axial T1-weighted postcontrast MR image with fat suppression. Only rim enhancement of the lesion, indicating the cystic nature of the lesion together with the T2-weighted image. Some soft tissue edema anteriorly, probably caused by a small cortical fissure. Radiologic images of case 5. D, A conventional radiograph of the right hip revealed a well-defined osteolytic lesion can be appreciated at the level of the lesser trochanter. There is a small fissure on the medial side. E, Conventional radiograph of the right hip acquired 3 years later after a fall. A fracture is visible through an osteolytic lesion which has increased in size compared with the previous radiograph. F, An axial T2-weighted MR image shows an expansion of the femur and a prominent fluid level. G, The postcontrast axial T1-weighted MR image with fat suppression demonstrates only enhancement at the rim of the lesion confirming the cystic nature of the lesion combined with the T2-weighted image. Some soft tissue edema on the lateral side caused by the fracture. Representative hematoxylin and eosin images of SBCs of different cases showing (h) fibrous septa with osteoid (H) and the typical “cloud-like” amorphous eosinophilic material (I). Reactive changes with signs of bleeding were present. J and K, Thickened fibrous septa were present between amorphous eosinophilic material and were more cellular with no atypia of the lining cells (L). M, Note the osteoclast-like giant cells.

Cases 3 to 7 were young boys (range between 8 and 15 y) presenting with a SBC in the femur (Fig. 4). In all cases, radiology showed an osteolytic, cystic lesion with fluid-fluid levels on MR with variable sizes and rim enhancement after contrast administration (Figs. 4A–G, Table 1). The radiologic differential diagnosis included a SBC and an ABC. In case 4, fibrous dysplasia was also considered. Histologically, all cases showed comparable morphology. Fibrous septa of variable thickness were encountered, which contained osteoid (Fig. 4H). Fragments of loose connective tissue with abundant eosinophilic “cloud-like” amorphous material were observed (Fig. 4I). Thin septations were lined by a layer of flattened cells, whereas thickened septa were more cellular, showed reactive changes with signs of bleeding and influx of inflammatory cells, and were lined by flattened to occasionally more enlarged protruding cells (Figs. 4J, K). Atypia and overt mitotic activity were not present.

Case 8 involved a 29-year-old female patient with a well-defined eccentric expansile osteolytic lesion of 2 cm in the proximal phalanx of the fourth finger of the left hand. On MR, the tumor was largely cystic with some strands of solid tissue. The morphologic evaluation revealed fragments of woven bone and abundant eosinophilic amorphous material. Focally, few septa were present with osteoclast-like giant cells and deposition of hemosiderin (Figs. 4L, M). Radiology and morphology were compatible with a SBC.

### Mapping of the Fusion to the Lining Cells as Well as the Surrounding Spindle Cells

**FIGURE 5:**
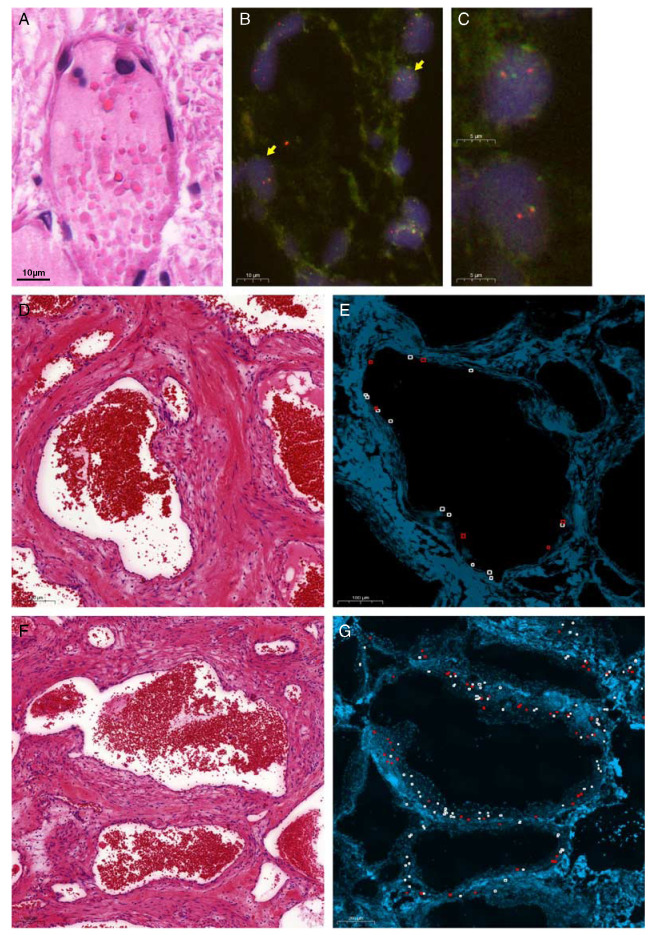
*EWSR1-NFATC2* translocation identified in the endothelial lining as well as the surrounding spindle cells in vascular malformation/hemangioma. *EWSR1-NFATC2* colocalization FISH (B, C) and corresponding area in hematoxylin and eosin section (A) in L6952 (case 1) highlights the presence of the fusion in the endothelial lining. Yellow arrows marking fusion cells. Scoring of *EWSR1-NFATC2* FISH in case 2 for the lining cells (D, E) and the surrounding spindle cells (F, G). Red boxes represent cells with colocalization of the EWSR1 and NFATC2 probe, while white boxes represent normal cells.

To identify the neoplastic cells in these benign lesions that were previously thought to be reactive, we carefully correlated the FISH results to the corresponding adjacent hematoxylin and eosin slides. In the vascular tumors, fusions were identified in both the endothelial lining cells as well as in the spindle cells in the stroma surrounding the vessels (Figs. 5A–C). We scored cells that line either the walls and the spindle cells in the stroma in various areas separately. In case 1, the fusion was much more common in the endothelial lining cells (27/37 nuclei, 73%) as compared with the surrounding spindle cells (15/42 nuclei, 35.7%), whereas in case 2, there was a more equal distribution (155/431 nuclei, 36% and 77/228 nuclei, 33.8%, respectively) (Figs. 5D–G). These findings suggest that in *EWSR1*-*NFATC2*-rearranged vascular malformation both the lining cells as well as the surrounding spindle cells are neoplastic. It was not possible to score lining cells and spindle cells separately in SBC.

### Immunohistochemistry

**FIGURE 6:**
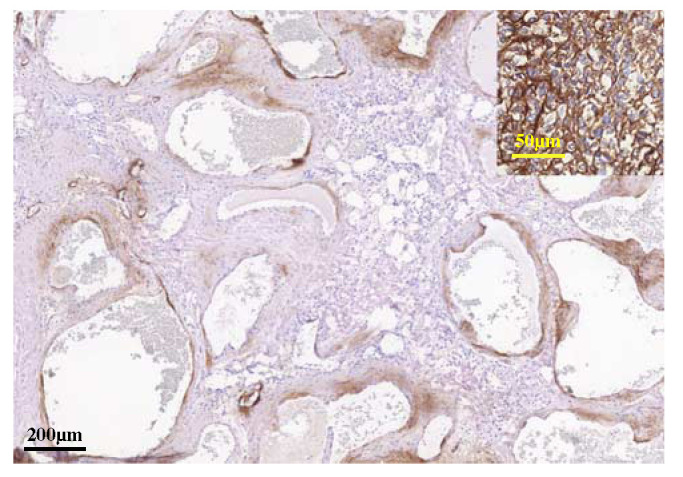
Immunohistochemistry for aggrecan (L6829). Expression is seen in the spindle cell areas surrounding most, but not all, of the spaces. Inset: *EWSR1-NFATC2*-rearranged round cell sarcoma as positive control.

To look for potential overlap in differentiation between the vascular tumors and SBC, immunohistochemistry using common endothelial markers CD31, CD34, and ERG was done (Table 2). As expected in SBC, vascular markers (CD31, CD34, and ERG) were negative; none of them displayed endothelial differentiation (Table 2). Interestingly, in the 3 vascular malformations, not all spaces were lined by cells expressing vascular markers; a mosaic pattern was seen in which some of the larger spaces with thicker walls were negative (Figs. 2H–J). On the basis of a recent publication indicating CD99, EMA, and SMA expression in SBC,^1^ we evaluated the expression of these markers in our series. While focal EMA expression was seen in SBC, this was absent in the 3 *NFATC2* rearranged vascular tumors. Expression of SMA and CD99 was more variable in both entities. NKX3-1 and NKX2-2, 2 recently reported markers for round cell sarcomas, including those with *EWSR1*-*NFATC2* fusion, were consistently negative in both vascular malformation and SBC in our cohort. Focal expression of aggrecan was found in all cases, and seemed to follow the mosaic pattern described above in the vascular malformations (Fig. 6). However, aggrecan staining was also observed in *EWSR1*-*NFATC2* negative vascular malformation (n=2) and SBC (n=2). Thus, aggrecan is not specific for *EWSR1*-*NFATC2*-rearranged tumors and cannot be used as a diagnostic marker in SBC and vascular malformation.

## DISCUSSION

**FIGURE 7:**
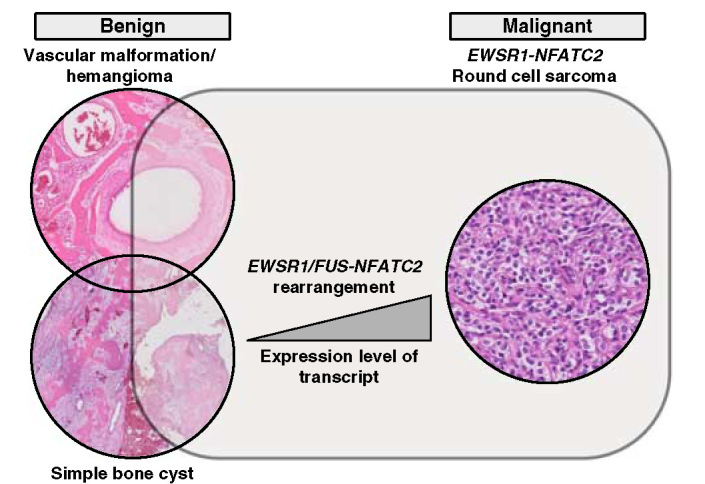
A proposed genetical relationship between *EWSR1-NFATC2* translocation carrying vascular malformation/hemangioma, SBC and round cell sarcomas. All entities carry the same genetic rearrangement but as *EWSR1-NFATC2* round cell sarcomas has amplification of the translocated genes an increased expression of the fusion transcript may contribute to the malignant behavior.

In addition to *EWSR1*-*NFATC2*-rearranged round cell sarcoma and SBC, identical fusions also occur in vascular malformations of bone (Fig. 7). Three vascular malformations of 2 patients revealed *EWSR1*-*NFATC2* rearrangements. Only 6 vascular malformations of 5 patients could be successfully analyzed, suggesting a frequency as high as 50%, but further studies are needed. The high failure rate is probably due to the prolonged tissue decalcification procedure which is required as these lesions usually cause excessive reactive bone formation. All of 6 conventional hemangiomas of bone were negative. Moreover, since we primarily focused on vascular tumors of bone, it would be interesting to see whether the fusions are also recurrent in soft tissue vascular tumors.

One vascular malformation patient (case 1) presented with multiple skeletal lesions, 2 of which were histologically analyzed, diagnosed as a benign vascular tumor, and confirmed to carry the *EWSR1-NFATC2* fusion. RNA analysis failed on one of them, so it was not possible to see whether both lesions had identical breakpoints in the fusion to confirm a clonal relation. The lesions were at distant sites (sacrum and rib, and probably also the skull) so it is unlikely that this reflects locoregional spread. Also, since both lesions did not show any sign of malignancy, it is also unlikely that they represent hematogenous metastases. It is well known that vascular tumors have a preference to affect multiple bones, which includes both malignant (epithelioid hemangioendothelioma) as well as nonmalignant (epithelioid hemangioma) tumors.^15^,^16^

Using careful mapping of the FISH results within the morphologic context, the *EWSR1*-*NFATC2* fusion was shown to be localized in the lining cells as well as in the spindle cells in the stroma surrounding the spaces, suggesting that both of these are neoplastic in vascular malformations. It is therefore tempting to hypothesize that translocation is an early event occurring in a progenitor cell such as the mesenchymal stem cells that can differentiate towards a stromal fibroblast as well as to an endothelial cell.^17^ In vitro mesenchymal stem cell can indeed differentiate to endothelial cells.^17^

The presence of a gene fusion indicates that these tumors are neoplastic and that the term “malformation” is actually a misnomer for these specific tumors. Therefore, in line with the fifth edition of the World Health Organization (WHO) classification of bone and soft tissue tumors, in which lesions previously referred to as arteriovenous malformation in soft tissue, are renamed as “arteriovenous malformation/hemangioma” based on the presence of *MAP2K1* gene mutations^3^ we propose to refer to the vascular lesions reported here as “vascular malformation/hemangioma with *EWSR1-NFATC2* rearrangement.”

Using AMP deep sequencing, FISH, and/or RT-PCR analysis, we also successfully analyzed 9 SBC, 6 of which demonstrated *NFATC2* rearrangement (66%). This is in line with previous reports on, in total, 18 cases of which 11 (~60%) showed *NFATC2* rearrangement.^1^,^2^ In our series, *EWSR1* was the most common fusion partner (at least 4/6) which is a bit higher as compared with previous reports where *FUS* was slightly more common (6/11) as compared with *EWSR1* (5/11).^1^,^2^ *NFATC2* fusions were absent in 6 tumors previously diagnosed as ABC, and in which the characteristic *USP6* fusion was lacking.^18^

There was limited morphologic overlap between the 3 vascular malformations/hemangiomas and SBC. The vascular malformations/hemangiomas demonstrated a mixture of thin-walled and thick-walled spaces of variable size. The amorphous eosinophilic cloud-like material that was frequently seen in the SBC cases, was absent in the vascular tumors. However, the lining cells in some of the cystic spaces in the vascular malformation/hemangioma, especially those displaying a thicker wall, lacked endothelial differentiation by immunohistochemistry, which was the only overlapping histologic feature between the SBC and the vascular malformation cases in our series. However, we cannot entirely rule out that this is artificial. Also, recently reported novel markers for *EWSR1-NFATC2*-rearranged round cell sarcoma such as NKX2-2 and NKX3-1^5^,^6^,^11^ were negative in the vascular malformations/hemangiomas with *EWSR1*-*NFATC2* rearrangement and SBCs. Staining of aggrecan was variably and sometimes extensively positive in the vascular tumors as well as in the SBCs. However, staining was also seen in cases that were negative for the *NFATC2* fusion, and hence it is not a good diagnostic marker.

While in SBC and in the 3 vascular malformations/hemangiomas with *EWSR1*-*NFATC2* rearrangement reported here morphologic features of malignancy are lacking, this is in sharp contrast to *NFATC2*-rearranged round cell sarcoma which are undifferentiated and clinically aggressive tumors, and thereby overtly malignant. Of interest, in round cell sarcomas, the *EWSR1*-*NFATC2* fusion is amplified, which is obvious when FISH analysis is done using probes flanking *EWSR1*.^4^ Of note, in nodular fasciitis, which is a benign mesenchymal tumor known to spontaneously regress, *USP6* rearrangement is a common finding, however, 2 cases with *USP6* rearrangement and amplification of the fusion are reported to show malignant behavior.^19^,^20^ Amplification of the fusion was absent in all cases of the current series. This suggests that in addition perhaps to the cell of origin, also the level of expression of the fusion transcript, determines tumor cell fate and biologic behavior. Amplification of the fusion in a cell may enhance its propensity for tumorigenesis. *EWSR1* is expressed in most cell types and has diverse roles in various cellular processes such as gene expression, meiotic and mitotic cell division.^21^ The N-terminal domain of EWSR1, which is usually retained during chromosomal translocation, was found to serve as a constitutive transcriptional activator to its fused partner.^22^,^23^ *NFATC2* is a transcription factor with documented roles in the immune system.^24^,^25^ Dysregulation of NFATC2 revealed various effects from cell cycle regulation to oncogenic functions. A study using breast-derived and colon-derived cell lines provided evidence that both NFATC2 and NFATC5 promote carcinoma invasion through α_6_β_4_ integrin dependent pathway.^26^ Interestingly, in vitro study of NFATC2 demonstrated an ability to activate MDM2 oncogene independent of TP53 through direct binding to its promoter and in turn reduces TP53 activation and functions.^27^ Moreover, overexpression of NFATC2 has been demonstrated to function as an oncogene by promoting the stemness of colorectal cancer stem cells through the Hippo pathway.^28^ The fusion leads to a truncation of the N-terminal part of the NFATC2 protein resulting in a loss of the regulatory elements and phosphorylation sites, while the EWSR1 partner adds a nuclear localization signal allowing a constitutional activation of the transcription factor.^4^ As such, amplification of the *NFATC2* fusion may result in the oncogenicity and aggressiveness of the tumor due to overexpression and gene dosage effect, however, the role of secondary changes involving any other coamplified gene cannot be ruled out. Therefore, further investigation is required to confirm this. A possible relationship between the level of *EWSR1/FUS-NFATC2* transcripts and the tumor spectrum identified in sarcomas is depicted in Figure 7.

Of interest, a hemangioma of bone with *EWSR1-NFATC2* rearrangement was reported previously in a 32-year-old man presenting with 2 intraosseous vascular lesions diagnosed as benign hemangioma.^29^ *NFATC1* is another member of the same gene family as *NFATC2* and the functional effects may be comparable. *EWSR1-NFATC2* rearrangements have not been reported in round cell sarcoma or SBC. While the vascular malformations/hemangiomas with *EWSR1-NFATC2* rearrangement reported here also show the presence of thick-walled vessels, for the *EWSR1-NFATC2* rearranged hemangioma the presence of only thin-walled vessels of varying caliber, embedded in a loose fibrous stroma is reported.^29^ However, no conclusions can be drawn as case numbers are very small.

It is not novel that identical gene fusions are found in different tumor entities. For instance, *EWSR1* or *FUS* can fuse to *ATF1* or *CREB1* in clear cell sarcoma of soft tissue as well as angiomatoid fibrous histiocytoma. Moreover, outside the soft tissues, these identical fusions can be found in hyalinizing clear cell carcinoma of the salivary gland (*EWSR1-ATF1*)^30^ or pulmonary myxoid sarcoma (*EWSR1-CREB1*).^31^ Clear cell sarcoma is a highly aggressive soft tissue sarcoma displaying melanocytic differentiation by immunohistochemistry, whereas angiomatoid fibrous histiocytoma is a rare neoplasm of intermediate (rarely metastasizing) malignant potential containing nodules of epithelioid to ovoid cells arranged in syncytial-like sheets.^3^ Similarly, *ETV6*-*NTRK3* gene fusions can be found in a wide range of tumor types, including congenital tumors (infantile fibrosarcoma and mesoblastic nephroma), secretory breast carcinoma, secretary carcinoma of the salivary gland, acute myeloid leukemia, and chronic eosinophilic leukemia.^32^–^37^

Thus, *EWSR1-NFATC2* rearrangement is another promiscuous gene fusion that can occur in a wide array of tumors: not only in malignant *NFATC2*-rearranged round cell sarcoma (with amplification of the fusion) and benign SBC (without amplification of the fusion), but now also in vascular malformation/hemangioma in elderly patients (Fig. 7).

## Supplementary Material

SUPPLEMENTARY MATERIAL

Supplemental Digital Content is available for this article. Direct URL citations appear in the printed text and are provided in the HTML and PDF versions of this article on the journal's website, www.ajsp.com.
