# Supplementary material for: Expanding the Spectrum of EWSR1-NFATC2-rearranged Benign Tumors: A Common Genomic Abnormality in Vascular Malformation/Hemangioma and Simple Bone Cyst
Source: Am J Surg Pathol. 2021 Jun 3;45(12):1669–81. doi: 10.1097/PAS.0000000000001748 (PMC8598111; doi:10.1097/PAS.0000000000001748)
Supplement: SUPPLEMENTARY MATERIAL [file pas-45-1669-s001.docx]

**Supp Table 1. Clinical and molecular features of all cases**

| **Case 1D.** | **Diagnosis** | **Gender/Age** | **Tumor location** | **Frozen Tissue** | **Archer (breakpoints)** | **FISH (Fusion**  **positive %)** | **RT-PCR**  **(EWSR1-NFATC2**  **Exon 6 – Exon 4)** |
| --- | --- | --- | --- | --- | --- | --- | --- |
| L4764 | ABC | F/19 | Humerus | Yes | NP | Failed | Negative |
| L5430 | ABC | F/14 | Tibia | Yes | no fusion | EWSR1-NFATC2 (0%) | Negative |
| L6192 | ABC | M/19 | Talus | Yes | NP | NFATC2 split  (0%) | Negative |
| L6827 | ABC | F/28 | Pelvis | Yes | no fusion | EWSR1-NFATC2 (3.7%) | Negative |
| L6853 | ABC | F/19 | Jaw (processus condylaris) | No | NP | EWSR1-NFATC2 (0%) | NP |
| L6856 | ABC | M/25 | Clavicle | No | no fusion | NFATC2 split  (17.9%) | NP |
| L6858 | ABC | M/8 | Vertebra L4 | No | NP | Failed | NP |
| L6830 | SBC | M/8 | Femur | Yes | EWSR1-NFATC2 (Exon 6 - Exon 3) | NFATC2 split (20.2%) | Negative |
| L6831 | SBC | M/15 | Femur | Yes | EWSR1-NFATC2 (Exon 6 - Exon 3) | Failed | EWSR1-NFATC2 (Exon 6 - Exon 3) |
| L6834 | SBC | M/27 | Pelvis | No | NP | Failed | NP |
| L6835 | SBC | M/13 | Jaw | No | no fusion | NFATC2 split  (8.1%) | NP |
| L6836 | SBC | M/13 | Femur | No | NP | NFATC2 split  (0%) | NP |
| L6837 | SBC | F/29 | Finger proximal phalanx dig 4 | No | no cDNA measured | NFATC2 split (51.2%) | NP |
| L6839 | SBC | M/14 | Humerus | No | NP | Failed | NP |
| L6840 | SBC | M/24 | Ulna | No | NP | Failed | NP |
| L6857 | SBC | M/9 | Femur | No | no cDNA measured | EWSR1-NFATC2 (12.3%) | NP |
| L6861 | SBC | M/9 | Femur | No | EWSR1-NFATC2 (Exon 6 - Exon 3) | EWSR1-NFATC2 (39.7%) | NP |
| L6863 | SBC | M/8 | Femur | No | no cDNA measured | NFATC2 split  (24.4%) | NP |
| L7186 | SBC | M/9 | Femur | No | EWSR1-NFATC2 (Exon 5 - Exon 3) | Failed | NP |
| L6829 | Vascular malformation | F/63 | Costa 5 anterior | Yes | EWSR1-NFATC2 (Exon 7 - Exon 3) | EWSR1-NFATC2 (49.4%) | EWSR1-NFATC2 (Exon 7 - Exon 3) |
| L6952 |  |  | Sacrum lesion | No | no cDNA measured | EWSR1-NFATC2 (53.2%) | NP |
| L6947 |  |  | Foot lesion | No | no cDNA measured | Failed | NP |
| L6530 | Vascular malformation | F/59 | Femur | Yes | NP | Failed | Negative |
| L6838 | Vascular malformation | F/42 | Os frontale (skull) | No | no fusion | NFATC2 split  (2.3%) | NP |
| L6855 | Vascular malformation | F/41 | Os frontale (skull) | No | NP | Failed | NP |
| L6862 | Vascular malformation | M/16 | Tibia | Yes | NP | Failed | NP |
| L6958 | Vascular malformation | M/3mo | Maxilla | No | no cDNA measured | EWSR1-NFATC2 (10.7%) | NP |
| L6959 | Vascular malformation | M/50 | Retrobulbar soft tissue | No | no cDNA measured | EWSR1-NFATC2  (36%) | NP |
| L6960 | Vascular malformation | M/59 | Spine, L2 | No | NP | failed | NP |
| L6961 | Vascular malformation | F/73 | Tibia | No | NP | Failed | NP |
| L4783 | Hemangioma of bone | F/53 | Vertebra Th4 | Yes | no fusion | NP | NP |
| L5163 | Hemangioma of bone | F/27 | Vertebra Th11 | Yes | no fusion | NP | NP |
| L5170 | Hemangioma of bone | F/56 | Costa 7 and 8 | Yes | no fusion | NP | NP |
| L5256 | Hemangioma of bone | F/31 | Vertebra Th10 | Yes | no fusion | NP | NP |
| L2997 | Hemangioma of bone | F/64 | Subperiosteum of tibia | Yes | no fusion | NP | NP |
| L6357 | Hemangioma of bone | M/22 | Vertebra Th7 | Yes | no fusion | NP | NP |
| L6569 | Hemangioma of bone | F/44 | skull | Yes | no fusion | NP | NP |

NP: not performed, highlighted: positive cases
