# Supplementary material for: Expanding the Spectrum of EWSR1-NFATC2-rearranged Benign Tumors: A Common Genomic Abnormality in Vascular Malformation/Hemangioma and Simple Bone Cyst
Source: Am J Surg Pathol. 2021 Jun 3;45(12):1669–81. doi: 10.1097/PAS.0000000000001748 (PMC8598111; doi:10.1097/PAS.0000000000001748)
Supplement: SUPPLEMENTARY MATERIAL [file pas-45-1669-s002.docx]

**Supp Table 2. Primary antibodies used for immunohistochemical staining**

| **Name** | **Clone** | **Catalog No.** | **Manufacturer** | **Antigen retrieval** | **Dilution** | **Species** | **Linker** | **Autostainer** |
| --- | --- | --- | --- | --- | --- | --- | --- | --- |
| AGGRECAN | - | ab186414 | Abcam | Citrate pH 6.0 | 1:16000 | rabbit | no | no |
| CD34 | QBEnd10 | GA632 | DAKO | Tris EDTA pH 9.0 | 1:1 | mouse | mouse | yes |
| CD99 | 12E7 | IR057 | DAKO | Tris EDTA pH 9.0 | 1:1 | mouse | no | yes |
| EMA | E-29 | GA629 | DAKO | Tris EDTA pH 9.0 | 1:1 | mouse | no | yes |
| ERG | EP111 | M7314 | DAKO | Tris EDTA pH 9.0 | 1:1000 | rabbit | rabbit | yes |
| NKX2-2 | 74.5 | 564731 | Pharmingen BD | Citrate pH 6.0 | 1:50 | mouse | mouse | yes |
| NKX3-1 | - | CP422B | Biocore medical | Tris EDTA pH 9.0 | 1:40 | rabbit | rabbit | yes |
| SMA | 1A4 | M0851 | DAKO | Tris EDTA pH 9.0 | 1:200 | mouse | no | yes |
